# Dietary cardoon meal modulates rumen biohydrogenation and bacterial community in lambs

**DOI:** 10.1038/s41598-021-95691-3

**Published:** 2021-08-10

**Authors:** Saheed A. Salami, Bernardo Valenti, Giuseppe Luciano, Massimiliano Lanza, Ngozi M. Umezurike-Amahah, Joseph P. Kerry, Michael N. O’Grady, Charles J. Newbold, Alessandro Priolo

**Affiliations:** 1 Department Di3A, Animal Production Science, University of Catania, Via Valdisavoia 5, 95123 Catania, Italy; 2 School of Food and Nutritional Sciences, College of Science, Engineering and Food Science, University College Cork, Cork, Ireland; 3 Dipartimento di Scienze Agrarie, Alimentari e Ambientali (DSA3), University of Perugia, Borgo XX Giugno 74, 06121 Perugia, Italy; 4 Scotland’s Rural College, Peter Wilson Building, King’s Buildings, Edinburgh, EH9 3JG UK

**Keywords:** Animal physiology, Microbial communities

## Abstract

Cardoon meal is a by-product of oil extraction from the seeds of *Cynara cardunculus* and can serve as a novel alternative feedstuff for ruminants. This study examined the rumen fermentation, biohydrogenation of fatty acids (FA) and microbial community in lambs fed a concentrate diet containing 15% dehydrated lucerne (CON, *n* = 8) or cardoon meal (CMD, *n* = 7) for 75 days pre-slaughter. Diets did not influence rumen fermentation characteristics and the abundance of bacteria, methanogens, fungi, or protozoa. Rumen digesta in CMD-fed lambs displayed a higher concentration of total saturated FA and lower total odd- and branched-chain FA and monounsaturated FA. Feeding CMD decreased total *trans*-18:1 isomer and the ratio of *trans*-10 to *trans*-11 C18:1, known as the “*trans*-10 shift”. Amplicon sequencing indicated that the rumen bacterial community in CMD-fed lambs had lower diversity and a higher relative phyla abundance of *Proteobacteria* at the expense of *Bacteroidetes* and *Fibrobacteres*. At the genus level, CMD mediated specific shifts from *Prevotella, Alloprevotella*, *Solobacterium* and *Fibrobacter* to *Ruminobacter*, suggesting that these genera may play important roles in biohydrogenation. Overall, these results demonstrate that cardoon meal can be used as a feedstuff for ruminants without negatively affecting rumen fermentation and microbiota but its impact on biohydrogenation may influence the FA composition in meat or milk.

## Introduction

The inclusion of agro-industrial by-products in animal diets is an alternative feeding strategy that could enhance the resource efficiency and sustainability of livestock production while reducing the economic cost and environmental burden associated with the disposal of these by-products^1^. Cardoon meal is a by-product obtained after the extraction of oil from the seeds of cultivated cardoon (*Cynara cardunculus* var. *altilis*), a perennial plant native to the Mediterranean region and widely distributed as a naturalized or invasive species in parts of Europe, the Americas and Oceania^2^. The global biomass of cardoon meal is increasing rapidly due to a renewed interest in the use of cardoon oil as an economical source of biodiesel^3,4^. Cardoon meal has been identified as a novel alternative feedstuff because of its potential as a valuable source of protein, amino acids, fiber and bioactive compounds such as unsaturated fatty acids (oleic and linoleic acids) and polyphenols^5–7^. Generally, limited research has been conducted to investigate the feeding potential of cardoon meal for poultry and ruminants^6–9^. In a recent in vitro fermentation study, Cabiddu et al.^6^ suggested that cardoon press cake (same as cardoon meal evaluated herein) could be used as a suitable feedstuff for ruminants without negative effects on ruminal digestion.

Fermentation of feed substrates in the rumen is accompanied by microbial lipolysis and subsequent ruminal biohydrogenation (RBH) of dietary unsaturated fatty acids (FA) resulting in the formation of saturated FA (SFA), *trans*-FA (TFA) and several FA intermediates^10^. Ruminant meat and milk are rich sources of healthy cis-monounsaturated FA (MUFA) and functional RBH intermediates, such as *trans*-11 18:1 and conjugated linoleic acids (CLA), with potential benefits on human health^11–13^. Bacteria are thought to be the most active microbes involved in RBH and recent advances in high-throughput sequencing could elucidate the diverse bacteria species involved in the complex biochemical pathways related to RBH intermediates. Species belonging to the *Butyrivibrio* genus (including *B. fibrisolvens*, *B. proteoclasticus*, *B. hungatei*) are possibly the largest group of bacteria involved in the hydrogenation and isomerization of linoleic and linolenic acids resulting in the formation of different *trans*-11 and *trans-*10 intermediates^14^. Additionally, bacteria strains belonging to the genera *Clostridium*, *Streptococcus*, *Staphylococcus*, *Lactobacillus*, *Propionibacterium*, *Pseudobutyrivibrio*, *Bifidobacterium*, *Eubacterium*, *Roseburia*, *Enterococcus* and *Pediococcus* have been implicated in the isomerization or hydration-dehydration process of converting unsaturated FA to CLA as an intermediate^14^. In a recent study, Zhang et al.^15^ showed that ruminal abundance of cellulolytic bacteria (*Ruminococcus albus*, *Ruminococcus flavefaciens*, *Fibrobacter succinogenes*, and *Butyrivibrio fibrisolvens*) in lambs was associated with higher conversion of polyunsaturated FA (PUFA) to SFA and concomitant accumulation of odd- and branched-chain fatty acids (OBCFA) and CLA. Despite the diversity of bacteria involved in RBH, this process seems to be species- and strain-specific, which complicates understanding the link between the rumen bacterial ecology and specific RBH intermediates in vivo^16^.

Nutritional strategies that increase the ingestion of unsaturated FA and polyphenols potentially modulate the rumen microbiome and inhibit RBH, and may consequently increase the accumulation of health-promoting unsaturated FA and CLA in meat or milk^13,17^. Therefore, it can be expected that bioactive compounds (polyphenols and unsaturated FA) present in dietary cardoon meal could exert functional effects on the rumen microbiome and metabolism to inhibit RBH and thus increase the ruminal outflow of unsaturated FA and CLA, and subsequent deposition in meat or milk. However, we reported in a companion paper that feeding cardoon meal induced specific changes in the intramuscular fatty acid profile of lambs by reducing the concentration of potentially health-promoting RBH intermediates (*trans*-11 C18:1 and *cis*-9, *trans*-11 CLA) and decreased the ratio of *trans*-10 18:1 to *trans*-11 18:1, known as the “*trans*-10 shift”^7^. As elucidated earlier, changes in RBH and rumen bacterial population could be the underlying mechanism for the observed differences in intramuscular fatty acids. Thus, it was hypothesized that residual phenolic compounds and unsaturated FA in cardoon meal might modulate the rumen microbiota and ruminal metabolism including alteration of RBH. The objective of this study was to investigate the effect of dietary cardoon meal on ruminal fermentation and RBH, and to utilize next-generation sequencing to characterize changes in the rumen bacterial community.

## Methods

### Animals, diets, slaughter, and rumen sampling

The experimental protocol was approved by the animal welfare and ethics committee of the University of Catania (FIR-2014-PI/LB/Di3A) and the feeding trial was conducted indoor at the experimental farm of the University as previously reported^18^. The animals were handled by specialized personnel according to the European Union legislation for the protection of animals used for scientific purposes (2010/63/ EU Directive) and the study was conducted in compliance with the ARRIVE guidelines (Animal Research: Reporting of In Vivo Experiments)^19^. Fifteen male Sarda × Comisana lambs (average age 75 ± 5 days and initial BW 19.58 ± 2.01 kg) were randomly assigned to two experimental groups. Each animal was reared in an individual pen and adapted to the experimental diets for a period of 9 d by progressive substitution of the weaning feed with the experimental feeds until a total replacement of the weaning diet was achieved. The control group (CON, *n* = 8), was raised on a commercial concentrate-based diet containing the following ingredients (*as-fed* basis): barley (48.0%), dehydrated lucerne (15.0%), wheat bran (23.0%), soybean meal (10.0%), molasses (2.0%) and vitamin premix (2.0%). The cardoon meal group (CMD, *n* = 7), received the same diet as the CON lambs except that the 15% dehydrated lucerne was completely replaced by cardoon meal. The concentrate pellets of both CON and CMD diets were formulated to meet the fiber and nutrient requirements of growing meat ruminants, calculated according to the Small Ruminant Nutrition System software (SRNS, version 1.11.7154.28131). The chemical composition of the cardoon meal and experimental diets are outlined in Table 1. The CON and CMD diets were supplied in form of pellets and lambs had ad libitum access to feeds and water for 75 days pre-slaughter. Diets were supplied daily, and the amount of refusal was measured before morning (09:00 h) feeding to calculate nutrient intakes from the total dry matter intake.

**Table 1:** Chemical composition of cardoon meal (CM) and experimental diets.

Parameter	Ingredient	Experimental diets^a^	
	CM	CON	CMD
Dry matter, % as fed	92.71	89.65	89.63
**Nutrient content**			
Crude protein, % DM	18.17	15.67	16.45
Ether extract, % DM	7.99	2.68	3.84
Ash, % DM	5.52	7.01	6.31
NDF, % DM	45.46	30.36	27.32
ADF, % DM	39.54	15.97	12.39
ADL, % DM	12.74	3.62	4.15
NFC	25.55	45.96	47.81
Total phenolic content^b^	60.39	5.21	13.08
**Protein fractions (% CP)**^**c**^			
A	6.05	10.34	5.65
B1	49.92	8.87	20.97
A + B1	55.97	19.21	26.63
B2	29.22	70.07	62.86
B3	5.72	7.02	7.23
C	9.08	3.70	3.28
**Fatty acids (mg/g DM)**^**d**^			
C14:0	0.06	0.03	0.03
C16:0	5.82	4.36	5.06
*cis*-9 C16:1	0.06	0.035	0.03
C18:0 SA^d^	1.51	0.45	0.71
C18:1 *n*-9 OA^d^	8.94	3.86	5.36
*cis*-11 C18:1	0.25	0.21	0.21
C18:2 *n*-6 LA^d^	28.03	12.19	16.85
C18:3 *n*-3 ALA^d^	0.07	1.26	1.07
C20:0	0.16	0.09	0.09

*NDF* neutral detergent fiber, *ADF* acid detergent fiber; *ADL* acid detergent lignin, *CP* crude protein, *NFC* non-fiber carbohydrates = 100 – ((NDF—NDFIP) + crude protein + ether extract + ash), where NDFIP represents the protein fraction linked to NDF.

^a^*CON* control diet; *CMD* cardoon meal diet.

^b^Expressed as grams gallic acid equivalents/kg DM.

^c^A: non-protein nitrogen; B1: true protein soluble in buffer; A + B1: total soluble nitrogen; B2: neutral detergent soluble protein; B3: neutral detergent insoluble protein but soluble in acid detergent; C: acid detergent insoluble protein.

^d^*SA:* stearic acid, *OA* oleic acid, *LA* linoleic acid, *ALA* α-linolenic acid.

The lambs were slaughtered (stunned by captive bolt before exsanguination) in a commercial abattoir, where they had free access to the experimental diets and water until approximately 3 h before slaughter. The pH of the ruminal digesta was measured immediately post-slaughter using a pH meter (Orion 9106, Orion Research Incorporated, Boston, MA). The entire ruminal digesta was collected from each lamb and thoroughly mixed, and two aliquots of the ruminal digesta (70–80 g) were collected within 20 min of slaughter and immediately placed in dry ice before storage at − 80 °C to preserve ruminal parameters for analysis of rumen fermentation, fatty acids and microbial population.

### Feed analysis

Cardoon meal and the experimental diet samples were analysed for chemical composition. Crude protein, ether extract and ash content were determined following the method of AOAC^20^. The neutral detergent fiber (NDF), acid detergent fiber (ADF) and acid detergent lignin (ADL) were analyzed as described by Van Soest et al.^21^. Phenolic compounds were extracted from the cardoon meal and experimental diets using aqueous methanol (50:50, vol/vol) and acetone (70:30, vol/vol) solvents^22^. Polyphenol-rich extracts were analyzed for total phenol content (TPC) using the Folin–Ciocalteu reagent. The TPC was expressed as g of gallic acid equivalent/g of dry matter (DM). Protein partitioning of cardoon meal and experimental diets into nitrogen fractions was carried out according to the Cornell Net Carbohydrate and Protein System (CNCPS) as described by Licitra et al.^23^. The FA composition in cardoon meal and the experimental diets was determined by a one-step extraction–transesterification procedure using chloroform^24^ and 2% (v/v) sulfuric acid in methanol^25^, with nonadecanoate (Larodan, Solna, Sweden) added as an internal standard. Gas chromatographic analysis of fatty acid methyl esters (FAME) was performed as described later for FA profile in the ruminal digesta. Individual FA was expressed as mg/g of DM.

### Analysis of ruminal fermentation characteristics

An aliquot of ruminal digesta from each lamb stored at − 80 °C was thawed overnight at 4 °C and used for VFA and ammonia analyses. The thawed aliquots were centrifuged at 1000×*g* for 20 min at 4 °C and 4 ml of the supernatant was added to 1 ml of 25% trichloroacetic acid (TCA) containing 20 mM 2-ethylbutyric acid as an internal standard. The VFA concentration was determined by gas–liquid chromatography as outlined by de la Fuente et al.^26^. Ammonia analysis was carried out by diluting the acidified rumen samples with 25% TCA in ratio 4:1, followed by centrifugation at 15,000×*g* for 15 min and the supernatant was analysed for ammonia concentration. The method described by Weatherburn^27^ was used to determine the ammonia concentration using a ChemWell^®^-T autoanalyser (Awareness Technology Inc., Palm City, FL, USA—Megazyme Cat. No. D-CHEMT). Microbial enzymatic activities (carboxymethyl cellulase, xylanase and amylase) of freeze-dried rumen samples were determined as described by Belanche et al.^28^. Lipase activity was determined by a spectrophotometric assay with *p-*nitrophenyl (PNP) butyrate as a substrate dissolved in acetonitrile at a concentration of 10 mM^29^. The specific activity of carboxymethyl cellulase, xylanase and amylase were expressed as milligram of sugar released per gram of protein per minute while lipase activity was expressed as millimolar PNP per gram of protein per minute.

### Analysis of ruminal fatty acid profile

Lipid was extracted in duplicate from 200 mg finely-ground freeze-dried ruminal digesta using a mixture of hexane and 2-propanol (3:2, vol/vol) as described by Shingfield et al.^25^. The extracted lipid was dissolved in 2 ml hexane and converted into fatty acid methyl esters (FAME) using a base-acid catalyzed transesterification procedure^30^. Sodium methoxide in methanol (0.5 M) and HCl (3 N) in methanol was used for base- and acid-catalyzed transesterification, respectively. The methyl esters were quantified on a gas chromatograph Trace Thermo Finningam GC equipped with a flame ionization detector (FID; ThermoQuest, Milan, Italy) and 100 m high polar fused silica capillary column (0.25 mm i.d., 0.25 μm, film thickness; SP 24056, Supelco, Bellefonte, PA). Helium was used as the carrier gas at a constant flow rate of 1 ml/min. Total FAME profile in a 2 µl sample volume at a split ratio of 1:50 was determined using the following GC conditions: oven temperature was programmed at 50 °C and held for 4 min, then increased to 120 °C at 10 °C/min, held for 1 min, increased up to 180 °C at 5 °C/min, held for 18 min, increased up to 200 °C at 2 °C/min, held for 15 min, and increased up to 230 °C at 2 °C/min, and held for 19 min. The injector and detector temperatures were at 270 °C and 300 °C, respectively. The identification of FAME was based on a standard mixture of 52 Component FAME Mix (Nu-Chek Prep Inc., Elysian, MN, USA) and 77 individual FAME standards (Larodan Fine Chemicals, Malmo, Sweden). The 18:1 and 18:2 isomers were identified based on commercial standard mixtures (Larodan Fine Chemicals) and chromatograms published by Kramer et al.^31^ and Alves and Bessa^32^. For individual FA, response factors to FID and inter-and intra-assay coefficients of variation were calculated by using a reference standard butter (CRM 164, Community Bureau of Reference, Brussels, Belgium). Fatty acids were expressed as g/100 g of total methylated fatty acids.

### DNA extraction and quantitative PCR assay

The extraction of DNA from freeze-dried ruminal digesta was performed using the cetyltrimethylammonium bromide (CTAB) detergent method^33^ except that cell lysis was achieved by incubation with sodium dodecyl sulphate (SDS) buffer for 10 min at 95 °C and potassium acetate was used for protein removal. The quantification of DNA yield was carried out by spectrophotometry (Nanodrop ND-1000 spectrophotometer). Extracted DNA samples were stored at − 80 °C prior to further analysis. DNA samples were quantified for total concentration of bacteria, anaerobic fungi, methanogens and protozoa using a LightCycler^®^ 480 System (Roche, Mannheim, Germany) as described by Belanche et al.^34^. Targeted primers used for quantitative PCR (qPCR) analysis of the microbes are indicated in Supplementary Table S1 online.

### Ion-torrent sequencing and data processing

The V1-V2 hypervariable region of 16S rRNA was amplified in extracted DNA samples for analysis of the bacterial community as described by Belanche et al.^35^. PCR amplification was carried out using targeted primers shown in Supplementary Table S1. The PCR amplicons were purified using Agencourt AMPure XP magnetic beads (Beckman Coulter Inc., Fullerton, USA) and DNA concentration was assessed using an Epoch Microplate Spectrophotometer (BioTek, Potton, UK). Further purification of amplicon library was carried out using the E-Gel Safe Imager Trans-illuminator with E-Gel Size Select 2% Agarose gels (Life Technologies Ltd., Paisley, UK). The purified DNA libraries were quantified for DNA yield and detection of artefacts post-PCR amplification using an Agilent 2100 Bioanalyzer with a high sensitivity DNA chip (Agilent Technologies, Ltd., Stockport, UK). The emulsion PCR of the DNA sample libraries was performed using the Ion PGM Template OT2 200 Kit (Life Technologies Ltd, Paisley, UK) following the appropriate manufacturer’s guide for users. Sequencing of the bacterial amplicon library was performed on the Ion Torrent PGM (Life Technologies Ltd, Paisley, UK) using the Ion PGM sequencing 316TM Chip v2.

The barcodes corresponding to individual samples were identified by multiplexing the sequences and low-quality datasets and chimeras were removed by denoising the sequences using MOTHUR software package^35^. The UPARSE pipeline (http://drive5.com/uparse/) was used to cluster the sequences into operational taxonomic units (OTU) at 97% identity^36^. The bacterial OTU table was normalized by random subsampling according to the sample with the minimum number of the sequence. The taxonomic classification of bacteria was carried out by comparison of the 16S rRNA gene sequences against the Ribosomal Database Project (RDP-II).

### Statistical analysis

Data on nutrient intakes, ruminal fermentation and FA profiles, and rumen microbial abundance were analyzed as a one-way ANOVA in SPSS (IBM Statistics version 22). Shapiro–Wilk normality test was applied to qPCR data and a log_10_ transformation was performed if unequal variances were found. Log-transformed data were subsequently analyzed by one-way ANOVA. Significance was considered when *P* < 0.05 and a tendency for treatment effect were observed when 0.05 < *P* ≤ 0.10. The biodiversity indices of the rumen bacterial community were calculated using the normalized OTU datasets using PRIMER-v6 software (PRIMER-E Ltd., Plymouth, UK). The dendrogram plot of hierarchical cluster analysis was generated using PRIMER-v6. The Vegan package in R statistical software (version 3.2.5; R Foundation for Statistical Computing, Vienna, Austria, https://www.R-project.org) was used to perform principal coordinate analysis (PCoA) on the log-transformed data using the mean Bray–Curtis distances and the multivariate analysis of variance (MANOVA) was used to assess the treatment significance. Heat maps and rarefaction curves were also constructed using the Vegan package in R statistical software. Treatment effect on the relative abundances of bacteria was analyzed using a one-way ANOVA as indicated for qPCR data. Correlations between the rumen bacterial community and ruminal FA profiles were elucidated on ordination plots using canonical correspondence analysis (CCA) generated in R statistical software. The significance level of the variables was computed using 999 random permutations.

## Results

### Diet composition and nutrient intakes

The chemical composition of experimental diets (CON and CMD) are outlined in Table 1. Dietary inclusion of cardoon meal enriched CMD with an approximately 2.5-fold greater concentration of phenolic compounds compared to CON containing dehydrated lucerne. The proportions of true soluble protein and total soluble nitrogen were 136.4% and 38.6% greater in CMD compared to CON. The inclusion of cardoon meal in CMD increased the concentration of oleic (+ 39.1%) and linoleic acids (+ 38.2%) compared to CON.

Feeding CMD reduced total dry matter intake (*P* = 0.013) and the intakes (*P* < 0.001) of neutral detergent fiber (NDF) (− 22.2%, *P* < 0.001), non-fiber carbohydrates (NFC) (− 10.1%, *P* = 0.013) and tended to decrease protein intake (− 9.3%, *P* = 0.078) compared to CON (Table 2). However, lambs fed CMD exhibited greater intakes (*P* < 0.05) of total fat and phenolic compounds compared to CON-fed lambs (Table 2). Dietary intake of individual fatty acids differed between the two animal groups. The CMD-fed lambs consumed greater amount of dietary 18:0 (*P* < 0.001), 18:1 *n*-9 (*P* = 0.005) and 18:2 *n*-6 (*P* = 0.006), and lesser amount of 18:3 *n*-3 (*P* < 0.001) than CON-fed lambs (Table 2).

**Table 2:** Nutrient intakes of lambs fed concentrate diets containing dehydrated lucerne (CON) or cardoon meal (CMD).

Nutrient intakes	Dietary treatment^a^		SEM	*P*-value
	CON	CMD		
Total DMI, g/day	1078.30	932.20	31.15	0.013
Total NDF intake, g/day	327.40	254.7	11.90	< 0.001
Total protein intake, g/day	168.97	153.34	4.450	0.078
Total phenol intake, g/day	5.62	12.19	0.917	< 0.001
Total fat intake, g/day	28.90	35.80	1.250	0.002
Total NFC intake, g/day	495.50	445.60	14.26	0.013
C16:0, g/day	4.70	4.72	0.117	0.934
C18:0 intake, g/day	0.49	0.66	0.027	< 0.001
C18:1 *n*-9 intake, g/day	4.16	5.00	0.164	0.005
C18:2 *n*-6 intake, g/day	13.14	15.71	0.507	0.006
C18:3 *n*-3 intake, g/day	1.35	0.99	0.055	< 0.001

*SEM* pooled standard error of the mean, *DMI* dry matter intake, *NDF* neutral detergent fiber, *NFC* non-fiber carbohydrate.

^a^*CON* control diet, *CMD* cardoon meal diet.

### Ruminal fermentation

Table 3 indicated that lambs fed CON and CMD diets displayed similar ruminal pH, total VFA concentration, and molar proportion of acetate, propionate, valerate and acetate/propionate ratio. However, the concentration of NH_3_-N tended to be greater (*P* = 0.086) in CMD-fed lambs. Dietary treatment did not affect the activities of carboxymethyl cellulose, xylanase, amylase and lipase measured in the rumen of lambs fed CON and CMD (Table 3).

**Table 3:** Rumen pH, fermentation traits and microbial enzyme activities in lambs fed concentrate diets containing dehydrated lucerne (CON) or cardoon meal (CMD).

Item	Dietary treatment^a^		SEM	*P*-value
	CON	CMD		
Rumen pH	6.50	6.54	0.125	0.869
NH_3_-N, mMol/l	13.67	19.82	1.790	0.086
Total volatile fatty acids (VFA), mM	55.15	52.56	7.956	0.878
Acetate	50.67	49.16	0.921	0.434
Propionate	32.73	32.76	1.180	0.991
Iso-butyrate	3.68	4.47	0.376	0.307
Butyrate	6.52	6.84	0.321	0.644
Iso-valerate	2.65	3.62	0.384	0.220
Valerate	3.75	3.16	0.317	0.364
Acetate/propionate ratio	1.59	1.52	0.074	0.662
**Microbial enzyme activities**^**b**^				
Carboxymethyl cellulase	34.92	30.85	4.151	0.643
Xylanase	129.15	119.61	16.599	0.786
Amylase	77.80	95.88	18.170	0.638
Lipase	2.46	2.13	0.253	0.545

*SEM* pooled standard error of the mean.

^a^*CON* control diet, *CMD* cardoon meal diet.

^b^Specific activity of carboxymethyl cellulase, xylanase and amylase were expressed as mg of sugar released per g of protein per min while lipase activity was expressed as mM PNP per g of protein per min.

### Ruminal fatty acid profile

**Figure 1:**
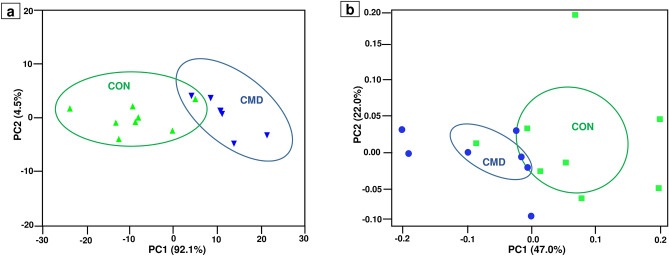
Principal coordinate analysis (PCoA) plot of **(A)** fatty acid profiles in rumen digesta **(B)** bacterial community structure in lambs fed the control diet (CON) and cardoon meal diet (CMD).

Fatty acid profile in the rumen digesta differed between dietary treatments when visualized on a PCoA plot (Fig. 1a). The fatty acid profiles of the ruminal digesta differed between lambs fed CON and CMD (Table 4). The CMD-fed lambs exhibited a greater concentration of total SFA (+ 41.3%; *P* < 0.001) than CON-fed lambs. The SFA concentration was dominated by C18:0 (stearic acid) which was greater (*P* < 0.001) in CMD-fed lambs. Also, the proportion of C16:0 was high in all lambs but there was no effect of diets on this FA. For differences in SFA with minor abundance, lambs fed CMD had a greater proportion of C20:0 (*P* < 0.05) compared to CON-fed lambs.

**Table 4:** Fatty acid composition of rumen digesta in lambs fed concentrate diets containing dehydrated lucerne (CON) or cardoon meal (CMD).

Item	Dietary treatment^a^		SEM	*P*-value
	CON	CMD		
**Fatty acids, g/100 g of total fatty acids**				
Σ SFA				
C4:0	0.49	0.36	0.060	0.279
C6:0	0.25	0.14	0.034	0.107
C8:0	0.16	0.11	0.016	0.122
C10:0	0.26	0.22	0.024	0.442
C12:0	0.40	0.35	0.047	0.592
C14:0	1.33	1.22	0.093	0.574
C16:0	12.90	12.62	0.275	0.622
C18:0	27.13	46.57	3.140	< 0.001
C20:0	0.52	0.66	0.031	0.023
C22:0	0.64	0.70	0.102	0.795
C24:0	0.53	0.46	0.038	0.398
ΣOBCFA				
C5:0	0.79	0.59	0.114	0.349
C9:0	0.28	0.24	0.023	0.448
C11:0	0.10	0.09	0.013	0.671
C13:0	0.20	0.15	0.024	0.259
*iso* C14:0	0.20	0.11	0.021	0.022
C15:0	0.90	0.67	0.064	0.069
*iso* C15:0	0.33	0.13	0.042	0.011
*anteiso* C15:0	1.29	0.60	0.107	< 0.001
*iso* C16:0	0.16	0.06	0.018	0.001
C17:0	0.51	0.37	0.046	0.148
*iso* C17:0	0.22	0.11	0.026	0.029
*anteiso* C17:0	0.40	0.20	0.060	0.111
C21:0	0.01	0.00	0.003	0.063
C23:0	0.59	0.32	0.070	0.057
Σ cis-MUFA				
*cis*-9 C12:1	0.21	0.14	0.021	0.102
*cis*-9 C14:1	0.18	0.16	0.017	0.494
*cis*-7 C16:1	0.13	0.13	0.018	0.980
*cis*-9 C16:1	0.47	0.40	0.067	0.644
*cis*-9 C17:1	0.08	0.03	0.013	0.054
*cis*-6 C18:1	1.22	0.87	0.085	0.035
*cis*-9 C18:1	5.45	4.26	0.313	0.053
*cis*-11 C18:1	0.94	0.78	0.067	0.251
*cis*-12 C18:1	1.12	0.92	0.084	0.226
*cis*-13 C18:1	0.13	0.11	0.021	0.694
*cis*-14 C18:1	0.28	0.29	0.051	0.964
*cis*-16 C18:1	0.41	0.39	0.061	0.932
*cis*-11 C20:1	0.28	0.20	0.027	0.149
*cis*-13 C22:1	0.21	0.10	0.020	0.001
ΣTrans-18:1				
*trans*-9 C14:1	0.08	0.01	0.019	0.076
*trans*-7 C16:1	0.04	0.03	0.007	0.438
*trans*-5 C18:1	0.30	0.22	0.039	0.330
*trans*-6 + 8 C18:1	1.68	0.75	0.173	0.003
*trans*-9 C18:1	1.02	0.70	0.067	0.009
*trans*-10 C18:1	9.43	1.94	1.290	0.001
*trans*-11 C18:1	1.59	0.98	0.154	0.046
*trans*-12 C18:1	0.42	0.38	0.033	0.506
Σ CLA				
*cis-9 trans*-11 C18:2	0.63	0.44	0.065	0.153
*trans*-8 *cis*-10 C18:2	0.05	0.06	0.010	0.560
*trans*-10 *trans*-12 C18:2	0.03	0.05	0.009	0.208
Σ AD				
*cis-9 trans*-12 C18:2	0.20	0.06	0.028	0.003
*trans*-8 *cis*-13 C18:2	0.08	0.06	0.018	0.560
*trans*-9 *cis*-12 C18:2	0.30	0.39	0.036	0.236
*trans*-9 *cis*-13 C18:2	0.13	0.08	0.013	0.056
*trans*-11 *cis*-15 C18:2	0.23	0.08	0.030	0.013
Σ LC-PUFA				
C18:2 *n*-6	5.10	5.06	0.539	0.976
C18:3 *n*-6	0.08	0.09	0.013	0.895
C18:3 *n*-3	0.39	0.25	0.043	0.104
C20:4 *n*-6	0.11	0.20	0.023	0.045
C20:5 *n*-3	0.32	0.23	0.037	0.264
C22:2 *n*-6	0.09	0.11	0.036	0.809
C22:4 *n*-6	0.08	0.06	0.013	0.590
C22:5 *n*-6	0.24	0.20	0.072	0.771
C22:5 *n*-3	0.03	0.03	0.007	0.688
C22:6 *n*-3	0.12	0.10	0.016	0.690
Summary				
Σ SFA	44.77	63.28	3.120	< 0.001
Σ OBCFA	5.98	3.65	0.390	< 0.001
Σ *trans*-18:1	14.44	4.97	1.600	< 0.001
*trans*-10/*trans*-11 18:1	5.98	2.85	0.794	0.044
Σ MUFA^b^	25.78	13.91	1.920	< 0.001
Σ PUFA^b^	8.21	7.56	0.651	0.635

*SEM* pooled standard error of the mean, *SFA* saturated fatty acids, *OBCFA* odd-and branched-chain fatty acids, *MUFA* monounsaturated fatty acids, *CLA* conjugated linoleic acids, *AD* atypical dienoic fatty acids, *PUFA* polyunsaturated fatty acids, *LC-PUFA* long-chain polyunsaturated fatty acids.

^a^*CON* control diet, *CMD* cardoon meal diet.

^b^Σ MUFA = Σ *cis*-MUFA + Σ *trans*-18:1; Σ PUFA = Σ CLA + Σ AD + Σ LC*-*PUFA.

Furthermore, dietary treatment affected the formation of OBCFA in the rumen (Table 4). Lambs fed CON accumulated greater concentration of total OBCFA (+ 39.0%; *P* < 0.001) compared to CMD-fed lambs. This alteration was mainly due to differences in the branched-chain FA. Lambs fed CON showed a greater (*P* < 0.05) abundance of *iso* C14:0, *iso* C15:0, *anteiso* C15:0, *iso* C16:0 and *iso* C17:0. Among the branched-chain FA, only *anteiso* C17:0 was not affected by diet. Regarding the odd-chain FA, there was a tendency to a greater accumulation of C15:0 (*P* = 0.069), C21:0 (*P* = 0.063) and C23:0 (*P* = 0.057) in CON-fed lambs.

Feeding CMD reduced the proportion of total MUFA (− 46.0%; *P* < 0.001), largely attributed to changes in total *trans* 18:1 isomers (− 65.6%, *P* < 0.001) (Table 4). Lambs fed CON displayed greater (*P* < 0.05) proportion of *trans*-6 + 8 C18:1, *trans-*9 C18:1, *trans*-10 C18:1 and *trans*-11 C18:1 compared to CON-fed lambs. Diet exhibited a minor effect on *cis*-MUFA; CMD decreased *cis*-6 C18:1 (*P* = 0.035) and *cis*-13 C22:1 (*P* = 0.001) and tended to lower *cis*-9 C17:1 (*P* = 0.054) and *cis*-9 C18:1 (*P* = 0.053).

There was no effect of diet on the concentration of total PUFA and long-chain PUFA such as C18:2 *n*-6, C18:3 *n*-3, C20:5 *n*-3, C22:5 *n*-3 and C22:6 *n*-3 (Table 4). However, the proportion of C20:4 *n*-6 (*P* = 0.045) was greater in animals fed CMD. Lambs fed CON and CMD had a similar proportion of CLA isomers (*cis*-9, *trans*-11 C18:2; *trans*-8, *cis*-10 C18:2 and *trans*-10, *trans*-12 C18:2). However, diet caused some differences in the proportion of atypical dienoic FA. Lambs fed CON showed greater percentage of *cis*-9, *trans*-12 C18:2 (*P* = 0.003) and *trans*-11, *cis*-15 C18:2 (*P* = 0.013) but tended to increase *trans*-9, *cis*-13 C18:2 (*P* = 0.056).

### Rumen microbiota

Data on quantitative PCR of microbial populations in the rumen are presented in Table 5. There was no effect of dietary treatment on the abundance of bacteria, methanogens, fungi and protozoa in the rumen.

**Table 5:** Rumen microbial numbers and bacterial diversity indices in lambs fed concentrate diets containing dehydrated lucerne (CON) or cardoon meal (CMD).

Item	Dietary treatment^a^		SEM	*P*-value
	CON	CMD		
**Microbial numbers**^b^				
Bacteria	8.83	8.82	0.034	0.869
Methanogens	6.63	6.18	0.205	0.276
Fungi	3.19	2.78	0.460	0.668
Protozoa	8.37	7.36	0.376	0.189
**Bacterial diversity indices**				
Number of OTU	441.25	436.57	32.316	0.946
Pielou’s evenness index	0.58	0.53	0.010	0.007
Shannon’s index	3.55	3.20	0.084	0.031
Simpson’s index	0.91	0.86	0.009	0.002

*SEM* pooled standard error of the mean, *OTU* operational taxonomic unit.

^a^*CON* control diet, *CMD* cardoon meal diet.

^b^Data were log-transformed to achieve normality and results expressed as log copy/mg DM of freeze-dried rumen content.

High-throughput sequencing of bacterial 16S rRNA genes generated approximately 600,000 raw sequences clustered into 2027 OTUs across 15 rumen samples. Normalization of the sequence datasets resulted in 318,857 high-quality sequences clustered into 2010 unique OTUs, averaging 21,257 sequences per sample. The plateau attained by the rarefaction curves (Supplementary Fig. S1) indicated that sampling of the rumen environment resulted in similar bacterial sequencing depth in lambs fed CON and CMD. Diet altered (MANOVA, *P* = 0.013) the rumen bacterial population structure when visualized on a PCoA plot (Fig. 1b). This observation was supported by the separate clustering of rumen samples from lambs fed CON and CMD on the dendrogram plot (Supplementary Fig. S2). The CMD-fed lambs exhibited a less even distribution of highly abundant and minor bacterial species (*P* < 0.05) and lower bacterial diversity values (Shannon’s index and Simpson’s index, *P* < 0.05) compared to CON-fed lambs.

The bacterial community in all samples was dominated by the phyla *Proteobacteria* (44.5%), *Bacteriodetes* (35.7%) and *Firmicutes* (14.3%) together with minor proportions of *Spirochaetes* (1.5%), *Actinobacteria* (0.5%), *Fibrobacteres* (2.6%) and unclassified sequences (0.6%) (Table 6). Furthermore, diet modulated the abundance of bacterial phylogenetic taxa. At the phylum level, lambs fed CMD had greater abundance of *Proteobacteria* (*P* < 0.01) and lower *Bacteriodetes* (*P* < 0.01) and *Fibrobacteres* (*P* < 0.05). This bacterial shift was obvious at the family taxa as CMD-fed lambs exhibited a greater abundance of *Succinivibrionaceae* (*P* < 0.05) and a decreased abundance (*P* < 0.05) of *Prevotellaceae* and *Fibrobacteraceae*. The distribution of bacterial genera in rumen samples presented few differences between diets as observed on the heat map (Supplementary Fig. S3). *Prevotella* and *Ruminobacter* are the most dominant genera in all animals and *Fibrobacter* appeared to be less abundant in CMD-fed lambs. The significance of the effect of diet on bacterial genera showed that CMD specifically increased the abundance of *Ruminobacter* (*P* < 0.01) and decreased *Fibrobacter* (*P* < 0.05). Moreover, CMD tended to decrease *Prevotella* (*P* = 0.070) and *Alloprevotella* (*P* = 0.096) in phylum *Bacteriodetes* and *Solobacterium* (*P* = 0.054) in phylum *Firmicutes*.

**Table 6:** Relative abundance (%) of rumen bacteria taxa (≥ 0.2% of average abundance) in lambs fed concentrate diets containing dehydrated lucerne (CON) or cardoon meal (CMD).

Phylum	Class	Family	Genus	Dietary treatment^a^		SEM	*P*-value
				CON	CMD		
Proteobacteria				37.72	51.25	2.775	0.009
	Gammaproteobacteria			37.02	50.80	2.879	0.011
		Succinivibrionaceae		34.51	46.76	2.567	0.011
			Ruminobacter	20.84	33.05	2.254	0.002
			Succinivibrio	0.16	0.50	0.198	0.416
			Anaerobiospirillum	6.20	2.01	1.596	0.201
	Alphaproteobacteria			0.50	0.17	0.185	0.404
Bacteroidetes				40.10	31.26	1.810	0.009
	Bacteroidia			39.02	30.31	1.822	0.011
		Porphyromonadaceae		3.14	3.56	0.408	0.625
			Barnesiella	2.75	3.04	0.421	0.746
		Prevotellaceae		31.38	22.57	2.150	0.035
			Prevotella	28.30	20.46	2.177	0.070
			Paraprevotella	0.51	0.30	0.108	0.350
			Hallella	0.45	0.26	0.105	0.405
			Alloprevotella	0.35	0.09	0.077	0.096
		Rikenellaceae		1.99	2.60	1.068	0.788
			Rikenella	1.99	2.60	1.068	0.788
Firmicutes				14.47	14.14	0.659	0.811
	Clostridia			6.74	6.49	0.459	0.791
		Ruminococcaceae		4.11	3.81	0.383	0.707
			Ruminococcus	1.40	0.99	0.228	0.386
			Acetivibrio	0.27	0.29	0.123	0.934
			Clostridium IV	0.15	0.29	0.115	0.547
		Lachnospiraceae		2.06	1.95	0.162	0.747
			Lachnospiracea incertae sedis	0.22	0.30	0.065	0.532
			Roseburia	0.89	0.54	0.139	0.221
			Butyrivibrio	0.39	0.27	0.082	0.509
	Negativicutes			4.69	5.42	0.299	0.237
		Veillonellaceae		1.81	2.34	0.326	0.433
			Dialister	0.93	0.69	0.256	0.647
			Mitsuokella	0.26	0.13	0.071	0.379
			Selenomonas	0.13	0.30	0.074	0.247
	Erysipelotrichia			2.89	1.76	0.452	0.226
		Erysipelotrichaceae		2.89	1.76	0.452	0.226
			Bulleidia	0.37	0.31	0.052	0.593
			Sharpea	0.71	0.86	0.265	0.798
			Solobacterium	0.55	0.08	0.124	0.054
			Kandleria	1.05	0.45	0.331	0.381
		Acidaminococcaceae		2.88	3.03	0.398	0.859
			Succiniclasticum	2.39	2.61	0.355	0.774
			Acidaminococcus	0.34	0.34	0.064	0.988
Spirochaetes				1.48	1.54	0.368	0.942
	Spirochaetia			1.48	1.54	0.368	0.942
		Spirochaetaceae		1.16	1.33	0.321	0.795
			Treponema	0.64	0.81	0.226	0.711
			Sphaerochaeta	0.49	0.33	0.136	0.584
Actinobacteria				0.49	0.44	0.118	0.823
	Actinobacteria			0.49	0.44	0.118	0.823
		Coriobacteriaceae		0.48	0.43	0.119	0.830
			Olsenella	0.47	0.42	0.120	0.839
Fibrobacteres				4.93	0.36	1.027	0.020
	Fibrobacteria			4.93	0.36	1.027	0.020
		Fibrobacteraceae		4.93	0.36	1.027	0.020
			Fibrobacter	4.93	0.36	1.027	0.020
Unclassified				0.47	0.71	0.101	0.238
	Unclassified			1.62	2.13	0.331	0.460
		Unclassified		7.70	8.56	0.999	0.685
			Unclassified	20.19	25.39	1.834	0.165

*SEM* pooled standard error of the mean.

^a^*CON* control diet, *CMD* cardoon meal diet.

**Figure 2:**
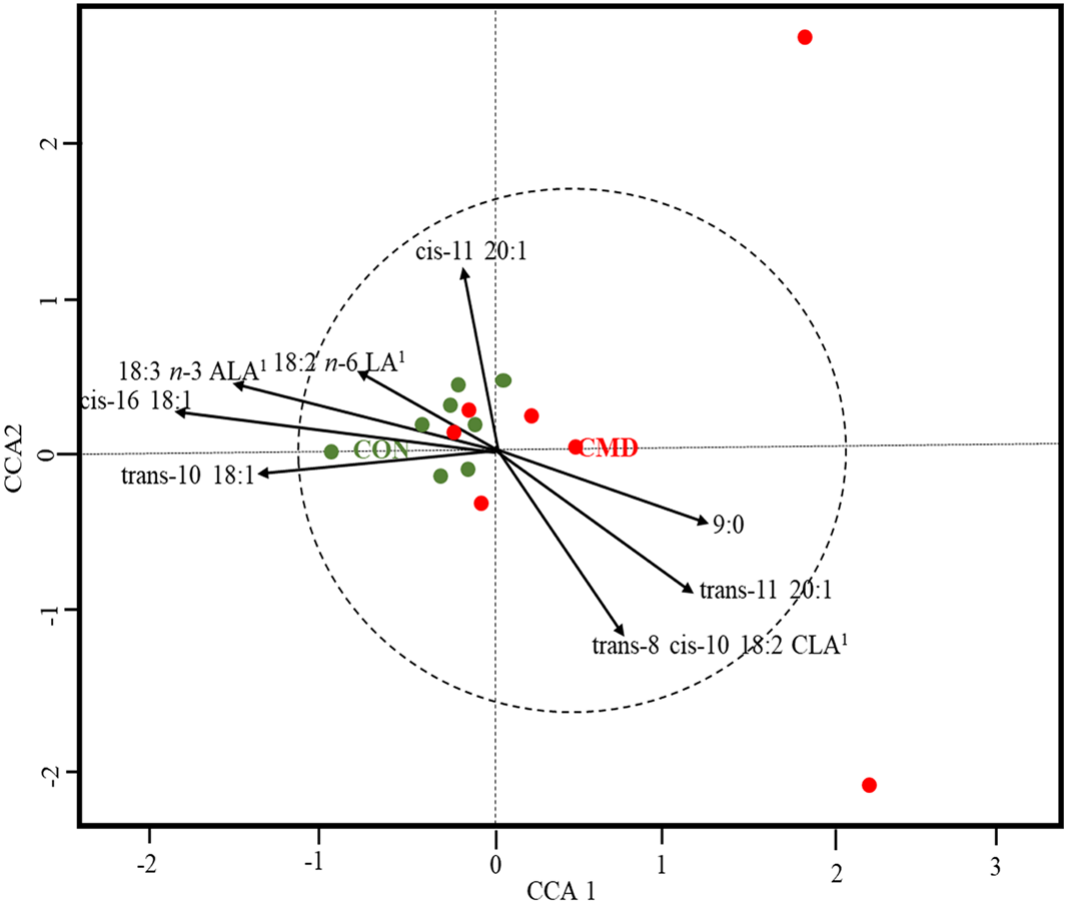
Canonical correspondence analysis (CCA) describing the correlations between the rumen bacterial community structure and the fatty acid profiles in rumen digesta. The arrows indicate the gradient direction, and their length is relative to the proportion of correlation. Arrows longer than the dotted circle signify correlation significance (*P* < 0.05). Colored dots represent the distribution pattern of lambs fed the control diet (CON) and cardoon meal diet (CMD). *LA* linoleic acid, *ALA* α-linolenic acid, *CLA* conjugated linoleic acid.

The correlation between the rumen bacterial community and ruminal FA profiles was elucidated on the CCA ordination plot (Fig. 2). There was a clear clustering between rumen samples from lambs fed CON and CMD. The bacterial community in CON-fed lambs positively correlated with *cis*-16 C18:1 (*P* = 0.009), *trans*-10 C18:1 (*P* = 0.038) and C18:3 *n*-3 (*P* = 0.009), with a tendency to correlate with C18:2 *n*-6 (*P* = 0.083) (Fig. 2 and Supplementary Table S2). In contrast, C9:0 (*P* = 0.085) and *trans*-8, *cis*-10 C18:2 (*P* = 0.081) tended to correlate with the bacterial community in CMD-fed lambs. Moreover, *cis*-11 C20:1 tended (*P* = 0.075) to correlate with the bacterial community but not distinctly related to either CON or CMD.

## Discussion

To our knowledge, the present study is the first to investigate the in vivo response of rumen function to dietary cardoon meal. In this study, lambs were fed a concentrate diet containing cardoon meal as a substitute for dehydrated lucerne. It was hypothesized that residual phenolic compounds and unsaturated FA in cardoon meal might modulate the rumen microbiota and ruminal metabolism including alteration of RBH. In agreement with previous data reported by Genovese et al.^5^, the nutritive value of cardoon meal is characterized by considerable amounts of protein, fiber, phenolic compounds, and unsaturated FA profiles. The predominance of oleic and linoleic acids in the FA profile of cardoon meal is consistent with the relative proportion of these FA in cardoon seeds^37^ and oil^38^.

Microbial fermentation of feed substrates in the rumen produces VFA and microbial proteins that supply ruminant animals with energy and metabolizable proteins, used for maintenance and production purposes^39^. In the present study, no effect of diet on rumen pH, total VFA, the molar proportion of individual VFA, microbial enzymatic activities nor abundance of rumen bacteria, methanogens, fungi and protozoa was observed. In agreement with this observation, cardoon meal did not alter in vitro VFA profile when incubated in ruminal fluid for up to 48 h^6^. Similarly, it has been shown that feeding up to 25% whole cardoon seeds did not impair rumen fermentation, nutrient digestibility and fibrolytic activity in sheep^37^. Regardless, the current study showed that replacing dehydrated lucerne with cardoon meal modified the structure of the rumen bacterial community without affecting ruminal fermentation. A possible explanation could be related to the fermentative redundancy of the rumen microbiome^40^, which allow the rumen ecosystem to maintain similar digestive function despite differences in microbial communities^41^. Moreover, subtle differences in unsaturated FA intakes are not expected to perturb ruminal feed degradability and fermentation as dietary fat level in this study did not exceed 6% diet DM that has been suggested as the threshold for fat concentration that could compromise rumen fermentation^42^.

Ruminal proteolysis influences the nitrogen utilization efficiency in ruminants with possible consequences for decreased animal performance and increased nitrogen emission into the environment^43^. Despite higher protein intake in lambs fed CON, the tendency for a greater concentration of ruminal NH_3_-N in CMD-fed lambs suggests that cardoon meal protein may be more susceptible to ruminal proteolysis compared to dehydrated lucerne protein. Ruminal NH_3_–N concentration is strongly influenced by the proportion of rumen-degradable protein fractions in the diet, with dietary soluble protein fraction rapidly metabolized to ammonia in the rumen^44^. A higher concentration of ruminal NH_3_–N was found in sheep fed up to 25% whole cardoon seeds, suggesting that cardoon seeds may contain a high amount of rumen-degradable protein^37^. In agreement with this assertion, we found a greater amount of total soluble nitrogen fraction in CMD compared to CON (Table 1), which may explain the substantial ruminal proteolysis resulting in higher NH_3_–N concentration in CMD-fed lambs.

Differences in diversity indices indicate that diet altered the taxonomic composition of the rumen bacterial community. Considering the crucial role of bacteria in ruminal digestion, the effect of diet on the bacterial community may influence other ruminal fermentation activities not measured in this study. The antimicrobial property of cardoon polyphenols^45^ could account for the inhibitory effect of CMD on the fibrolytic bacterium (*Fibrobacter*), which may negatively affect ruminal fiber digestion when a high-fiber diet is fed^46^. Furthermore, the higher abundance of *Proteobacteria*, predominantly *Succinivibrionaceae*, in CMD-fed lambs may be linked to reduced CH_4_ production in the rumen. Previous studies have consistently shown that the dominance of *Proteobacteria* belonging to *Succinivibrionaceae* is closely associated with low CH_4_ emission in dairy cows^47^, beef cattle^48^ and Tammar wallabies^49^. Thus, it would be interesting for future studies to investigate the effect of dietary cardoon meal on other ruminal fermentation activities such as nutrient digestibility and methanogenesis.

Ruminal biohydrogenation of unsaturated FA to SFA reduces the outflow of health-promoting unsaturated FA from the rumen for subsequent absorption and incorporation into ruminant meat or milk. However, RBH also produces several intermediates (mainly C18:1 and C18:2 isomers) including *trans*-11 C18:1 and *cis*-9, *trans*-11 CLA that exhibit potential health benefits in humans when incorporated in ruminant-derived foods^50^. Cabiddu et al.^6^ reported that plant secondary metabolites (phenolics and polyphenol oxidase) present in cardoon meal (32.7 mg tannic acid equivalent/g DM) could be responsible for the reduction in RBH of linoleic and linolenic acids observed in an in vitro ruminal study. In the present study, dietary cardoon meal increased the intake of unsaturated FA (linoleic and oleic acids) and phenolic compounds as expected. However, the extent of RBH was greater in CMD-fed lambs as evident with greater accumulations of stearic acid, SFA, and lower concentrations of oleic acid, *trans*-11 C18:1 and MUFA. This observation contradicts the expectation that high phenolic content in cardoon meal could reduce RBH compared to dehydrated lucerne. It is noteworthy that differences in unsaturated FA intakes found in this study are comparable to those reported in other studies where reduced RBH have been demonstrated due to the interaction with dietary polyphenol content from pomegranate by-product (18.9 g tannic acid equivalents (TAE)/kg DM)^51^ and hazelnut skin (22.4 g TAE/kg DM)^52^. Moreover, recent reviews of the scientific literature have shown that dietary polyphenols can be effective to reduce RBH in basal diets supplemented with or without PUFA-rich sources^53,54^. However, the inhibition of RBH by dietary phenolic compounds is inconsistent across studies due to the interaction of factors such as structural complexity, type of diets, level of polyphenol intake, animal species and physiological status, as well as characteristics inherent to the basal diet^55^.

Triglycerides (TG), phospholipids (PL) and glycolipids (GL) are the main forms of dietary lipids entering the rumen. The TG are the dominant lipids in cereals and oilseeds (including vegetable oil and oilseed by-products such as cardoon meal) while the main lipid fractions in forages (such as lucerne) are PL and GL, which are usually associated with cellular membranes^13^. The PUFA esterified to these complex lipids have to be hydrolyzed to free fatty acids through lipolysis as a prerequisite for subsequent occurrence of RBH. Lashkari et al.^56^ recently showed that PUFA esterified to PL are less prone to lipolysis and RBH than those esterified to TG. This suggests that differences between the lipid fraction composition of lucerne (PL + GL) and cardoon meal (TG) could partly explain the reduced RBH in CON-fed lambs compared to those fed CMD. Additionally, there could be an interaction between dietary lipids and polyphenols when plant esterified lipids are inherently localised within protein-phenol complexes, making the lipid substrate unavailable for lipolytic enzyme activity and therefore reducing ruminal lipolysis and RBH^57^. This lipid-phenol interaction might be more prominent in forages (such as lucerne), which may also explain the lower RBH^57,58^.

Furthermore, the total phenol intake of lambs fed CMD (i.e., 12.2 g/day) is within the range of values reported in studies where carob pulp (12.0–13.4 g/day)^59^, pomegranate by-product (16.7 g/day)^51^ and hazelnut skin (19.1 g/day)^52^ were included in lamb concentrate diets that have been shown to reduce RBH as reflected by the FA profile of ruminal digesta and muscle. This suggests that the lack of inhibitory effect of cardoon meal on RBH is unlikely to be related to the level of phenol intake. Thus, the discrepancy between our results and that of other studies with similar phenol intake could be partly due to possible differences in phenolic compound composition. Tannins are the predominant polyphenols in carob pulp^59^, pomegranate by-product^51^ and hazelnut skin^52^ whereas the main phenolic compounds present in cardoon morphological parts (leaf, seed, stem and flower) are flavonoids (luteolin, apigenin, quercetin etc.) and caffeoylquinic acid derivatives, of which chlorogenic acid and *trans*-cinnamic acid are abundant^60–62^. Indeed, there is limited information on the efficacy of cardoon phenolic compounds in inhibiting RBH. It has been shown that hydroxycinnamic acids including caffeic and chlorogenic acids can be metabolised by the gut microflora^63^. This suggests that the phenolic compounds present in CMD might have been metabolised by the rumen microbiota, resulting in a lack of efficacy of CMD in inhibiting RBH. In another study, dietary addition of quercetin (250 and 500 mg/l) or triterpene saponin extracts (500 and 1000 mg/l) did not inhibit RBH assessed in dual-flow continuous culture fermenters^64^. Moreover, extract from the flower leaves of the artichoke (*Cynara scolymus* L.), a botanical relative of cultivated cardoon, exhibited high polyphenol oxidase activity but was not effective in inhibiting in vitro RBH of α-linolenic acid^65^. In contrast, the presence of saponins and flavonoids in lucerne may contribute to its greater inhibitory effect on in vitro RBH compared to phenolic-rich plant species such as birdsfoot trefoil, chicory and English plantain^66^. Moreover, heat treatment of feedstuffs, as applied in the production of dehydrated lucerne, causes changes in the protein matrix surrounding the fat droplets resulting in a simultaneous reduction in ruminal proteolysis and RBH^67,68^. This theory is consistent with a tendency for reduced ruminal proteolysis and RBH inhibition found in lambs fed CON compared to CMD-fed lambs. The inhibitory effect of dietary lucerne on RBH may account for increased PUFA and CLA contents in ruminant milk and meat^69,70^. Thus, it would be interesting for future studies to investigate the application of feed processing methods such as heat treatment to protect proteins and unsaturated FA in cardoon meal against ruminal proteolysis and RBH, respectively.

Bacteria are thought to be the main microbes responsible for RBH but there is limited knowledge on the complexity of bacterial species related to the production of several RBH intermediates. In this study, high-throughput sequencing of 16S rRNA genes revealed that changes in the rumen bacterial community could be the underlying mechanism responsible for observed differences in RBH. The bacterial shift towards *Proteobacteria*, mainly *Succinivibrionaceae*, could account for the substantial RBH in CMD-fed lambs. This is consistent with previous studies that linked the abundance of *Succinivibrionaceae* to increased RBH in cattle, sheep and goats fed with dietary lipid supplements^71,72^. However, it appears that the present study is the first to classify that increased abundance of family *Succinivibrionaceae* specifically occurs in genus *Ruminobacter*. Moreover, increased RBH in CMD-fed lambs was associated with a lower abundance of *Prevotella, Alloprevotella, Solobacterium* and *Fibrobacter*. In agreement with our observation, Huws et al.^73^ indicated that decreased abundance of *Prevotella* and *Fibrobacter* correspond to substantially increased RBH in the rumen of cattle fed a grass silage diet supplemented with flax oil. This suggests that these bacterial genera may be preferentially suppressed at the expense of bacteria (e.g. *Succinivibrionaceae*) involved in complete RBH of unsaturated FA. Furthermore, a recent study by Zhang et al.^15^ showed that ruminal accumulation of OBCFA (C15:0, *iso* C14:0, *iso* C15:0, *anteiso* C15:0 and *anteiso* C17:0) was associated with the abundance of cellulolytic bacteria including *Fibrobacter succinogenes* and *Prevotella brevis* in lambs. In agreement with our study, we found a similar increase in OBCFA in the rumen digesta of CON-fed lambs, accompanied by a higher abundance of *Fibrobacter* and *Prevotella* genera, confirming that these bacterial groups could be involved in the synthesis of OBCFA intermediates.

Feeding CON inhibited the terminal step of RBH as indicated by a lower concentration of C18:0 and a greater proportion of total *trans* 18:1 isomers including *trans*-11 C18:1 and *trans*-10 C18:1. *Trans*-11 C18:1 is the main precursor desaturated into *cis*-9, *trans*-11 CLA in the muscle tissues or mammary gland, known for its potential human health benefits when incorporated into meat or milk^74^. On the other hand, increased concentration of ruminal *trans*-10 C18:1 resulting in “*trans*-10 shift” has been implicated in the modification of mammary lipogenesis, causing a reduction of milk fat synthesis in dairy ruminants^75^. Considering that “*trans*-10 shift” is postulated to have occurred when the ratio of *trans*-10/*trans*-11 18:1 > 1^76^, the present results suggest that both CON and CMD induced “*trans*-10 shift” but this occurs to a greater extent in CON-fed lambs. Interestingly, this pattern of FA changes in the rumen digesta is consistent with the observations found in the intramuscular fatty acid profile as reported in a companion paper^7^. The occurrence of *trans*-10 shift has been associated with different factors, including high starch content in concentrate-based diets that could induce changes in rumen pH and fermentation^76,77^. Given that starch is the dominant component of NFC fraction in concentrate diets^78^, a higher NFC intake in CMD-fed lambs could be an indication of greater starch intake. However, the discrepancy in NFC intake did not induce changes in rumen pH and fermentation traits in this study. Therefore, it is unlikely that potential difference in starch intake is sufficient to explain why the extent of the *trans*-10 shift was greater in lambs fed CON compared to those fed CMD.

Other possible mechanisms for the discrepancy in “*trans*-10 shift” could be related to the occurrence of alternative RBH pathways for the formation of CLA isomers, *trans*-10 and *trans*-11 C18:1. Feeding concentrate-based diets induced the accumulation of *trans*-10 C18:1 in the rumen^75^ but it is unclear if the formation of *trans*-10 C18:1 occurs as a competing intermediate to *trans*-11 C18:1^76^. In this study, the simultaneous accumulation of *trans*-11 18:1 and *trans*-10 18:1 in CON-fed lambs suggests that the production of both intermediates might have occurred via different RBH pathways^79^. Differences in the lipid fraction of lucerne (PL + GL) and cardoon meal (TG) and their esterified PUFA composition could influence the formation of CLA isomers and mediate alternative pathways for the RBH of linoleic and linolenic acids^56^. A plausible explanation may relate to the fact that ruminal digesta in CON-fed lambs contained a greater proportion of *trans*-11, *cis*-15 18:2, a major non-conjugated 18:2 isomer from which *trans*-11 18:1 could be formed via an alternative pathway for α-linolenic acid hydrogenation^80^. On the other hand, *trans*-10 18:1 could be formed via isomerisation of oleic acid or reduction of *trans*-10, *cis*-12 18:2^75^. The reduction pathway is unlikely in the present study because *trans*-10, *cis*-12 18:2 was not identified in the rumen of lambs. However, a greater concentration of oleic acid in CON-fed lambs could enable an isomerisation pathway to produce *trans*-10 18:1.

Bacterial strains belonging to *Butyrivibrio* spp. are believed to be mainly responsible for the hydrogenation of unsaturated FA (linoleic and α-linolenic acids) via C18:2 intermediates to *trans*-11 C18:1 and eventually to C18:0^81^. However, the abundance of *Butyrivibrio* spp. was not affected in this study, in agreement with recent molecular studies that linked other several as-yet-uncultured bacteria to RBH^71,72^. The CCA ordination plot indicated that the rumen bacterial community in CON-fed lambs favoured the accumulation of α-linolenic acid and *tran*s-10 C18:1, and tended to promote oleic acid concentration, suggesting a possible link of the bacterial community with incomplete RBH. In comparison to CMD-fed lambs, the rumen of lambs fed CON was characterized by a lower abundance of *Succinivibrionaceae* (*Ruminobacter*) and a greater abundance of *Prevotellaceae* (*Prevotella*, and *Alloprevotella*), *Solobacterium* and *Fibrobacteraceae* (*Fibrobacter*). However, there is limited knowledge on the metabolic role of these bacterial groups in RBH.

## Conclusions

The inclusion of cardoon meal to replace dehydrated lucerne in a concentrate diet resulted in differences in nutrient intakes without affecting ruminal fermentation characteristics in lambs. However, dietary inclusion of cardoon meal mediated changes in RBH that may reduce the accumulation of health-promoting unsaturated FA (MUFA and *trans*-11 C18:1) in ruminant meat or milk. The changes in RBH could explain the underlying mechanism of specific differences such as lower *trans*-11 C18:1, *cis*-9, *trans*-11 CLA and *trans*-10/*trans*-11 18:1 ratio observed in previously reported intramuscular fatty acid profile. The effect on RBH was accompanied by a modification of the rumen bacterial community. Feeding CMD mediated a specific shift from *Prevotella*, *Alloprevotella, Solobacterium* and *Fibrobacter* to *Ruminobacter*. Future studies should investigate the role of these bacteria genera in RBH to enhance the understanding of strategies required for manipulating specific bacteria groups related to the formation of important RBH intermediates such as *trans*-11 C18:1 and *trans*-10 C18:1. Moreover, further investigation is required to understand if the effect of dietary cardoon meal on the rumen bacterial ecology could affect other ruminal activities including nutrient digestibility and methanogenesis.

## Supplementary Information

Supplementary Information.

## Data Availability

Raw sequence reads from the bacterial library was deposited at the EBI Sequence Read Archive (SRA) of the European Nucleotide Archive and can be accessed under the study accession number: PRJEB36716. The data that support the findings of this study are available from the corresponding author upon reasonable request.
