# Supplementary material for: Dietary cardoon meal modulates rumen biohydrogenation and bacterial community in lambs
Source: Sci Rep. 2021 Aug 10;11:16180. doi: 10.1038/s41598-021-95691-3 (PMC8355377; doi:10.1038/s41598-021-95691-3)
Supplement: Supplementary file 1 — Supplementary Information. [file 41598_2021_95691_MOESM1_ESM.pdf]

Dietary cardoon meal modulates rumen biohydrogenation and bacterial community in lambs

Saheed A. Salami[1,2], Bernardo Valenti[1], Giuseppe Luciano[1], Massimiliano Lanza[1], Ngozi M. Umezurike-Amahah[1], Joseph P. Kerry[2], Michael N. O'Grady[2], Charles J. Newbold[3] and Alessandro Priolo[1]*

[1]*Department Di3A, Animal Production Science, University of Catania, Via Valdisavoia 5, 95123 Catania, Italy*

[2]*School of Food and Nutritional Sciences, College of Science, Engineering and Food Science, University College Cork, Cork, Ireland*

[3]*Scotland's Rural College, Peter Wilson Building, King's Buildings, EH9 3JG, Edinburgh, United Kingdom*

[*]Corresponding author: s.salami@umail.ucc.ie

Supplementary Table S1. List of primers used for quantitative PCR and ion torrent next generation sequencing (NGS)

Target gene and group	References	Primer set	Sequence (5` to 3` direction)	T[a]	Amplicon (bp)
Quantitative PCR					
18S rDNA for total protozoa	Sylvester, et al. [1]	PSSU-316f<br>PSSU-539R	F: GCTTTCGWTGGTAGTGTATT<br>R: CTTGCCCTCYAATCGTWCT	55	223
16S rRNA for total bacteria	Maeda, et al. [2]	Univ16S_1048-1067<br>Univ16S_1175_1194	F: GTGSTGCAYGGYTGTCGTCA<br>R: ACGTCRTCCMCACCTTCCTC	61	150
[a]*mcrA* gene for methanogenic archaea	Denman, et al. [3]	qmcrA-f<br>qmcra-r	F: TTCGGTGGATCDCARAGRGC<br>R: GBARGTCGWAWCCGTAGAATCC	56	140
18S rRNA and ITS1 for total anaerobic fungi	Denman and McSweeney [4]	Denfun f<br>Denfun r	F: GAGGAAGTAAAAGTCGTAACAAGGTTTC<br>R: CAAATTCACAAAGGGTAGGATGATT	62	120
Ion torrent NGS					
Bacterial Adaptors			F: CCATCTCATCCCTGCGTGTCTCCGACTCAG<br>R: CCTCTCTATGGGCAGTCGGTGAT		
Bacterial primers	Spear, et al. [5]	27F<br>357R	F: AGAGTTTGATCMTGGCTCAG<br>R: CTGCTGCCTYCCGTA	58	348

[a]mcrA gene: methyl-coenzyme M reductase alpha subunit gene

Supplementary Table S2. Correlation coefficients of canonical correspondence analysis indicating relationship between rumen bacterial community structure, fermentation and microbial variables

Item	R^2	*P*-value
4:0	0.034	0.847
5:0	0.144	0.398
6:0	0.039	0.649
8:0	0.015	0.924
9:0	0.351	0.085
10:0	0.226	0.313
11:0	0.107	0.567
12:0	0.150	0.480
cis-9 12:1	0.020	0.866
13:0	0.039	0.530
14:0	0.212	0.286
cis-9 14:1	0.097	0.675
trans-9 14:1	0.058	0.695
iso 14:0	0.060	0.689
15:0	0.015	0.913
iso 15:0	0.080	0.718
anteiso 15:0	0.049	0.762
16:0	0.029	0.769
cis-7 16:1	0.236	0.190
cis-9 16:1	0.090	0.728
trans-7 16:1	0.227	0.327
iso 16:0	0.114	0.639
17:0	0.092	0.301
cis-9 17:1	0.116	0.462
iso 17:0	0.046	0.745
anteiso 17:0	0.232	0.226
18:0 SA[1]	0.181	0.328
cis-6 18:1	0.168	0.397
18:1 *n*-9 OA[1]	0.233	0.338
cis-11 18:1	0.066	0.774
cis-12 18:1	0.101	0.632
cis-13 18:1	0.182	0.443
cis-14 18:1	0.333	0.127
cis-16 18:1	0.544	0.009
trans-5 18:1	0.124	0.634
trans-6+8 18:1	0.294	0.165
trans-9 18:1	0.0128	0.966
trans-10 18:1	0.366	0.038
trans-11 18:1 VA[1]	0.171	0.464
trans-12 18:1	0.133	0.416
18:2 *n*-6 LA[1]	0.317	0.083
cis-9 trans-11 18:2 CLA	0.003	0.943
cis-9 trans-12 18:2	0.201	0.267
trans-8 *cis*-10 18:2 CLA	0.356	0.081
trans-8 *cis*-13 18:2	0.001	0.990
trans-9 *cis*-12 18:2	0.009	0.968

Supplementary Table S2 (continued).

trans-9 *cis*-13 18:2	0.128	0.611
trans-10 *trans*-12 18:2 CLA	0.200	0.229
trans-11 *cis*-15 18:2	0.111	0.510
18:3 *n*-6	0.067	0.761
18:3 *n*-3 ALA[1]	0.458	0.022
20:0	0.043	0.803
cis-11 20:1	0.334	0.075
trans-11 20:1	0.456	0.091
20:4 *n*-6	0.255	0.234
20:5 *n*-3 EPA[1]	0.060	0.642
21:0	0.084	0.386
22:0	0.270	0.195
cis-13 22:1	0.265	0.235
22:2 *n*-6	0.056	0.789
22:4 *n*-6	0.137	0.469
22:5 *n*-6	0.029	0.94
22:5 *n*-3 DPA[1]	0.007	0.971
22:6 *n*-3 DHA[1]	0.203	0.341
23:0	0.098	0.577
24:0	0.078	0.490
Σ SFA	0.195	0.258
Σ MUFA	0.291	0.146
Σ PUFA	0.302	0.107
Σ OBCFA[2]	0.086	0.751
Σ trans 18:1	0.272	0.191

[1]SA: stearic acid; OA: oleic acid; LA: linoleic acid; ALA: α-linolenic acid; EPA: eicosapentaenoic acid; DPA: docosapentaenoic acid; DHA: docosahexaenoic acid; OBCFA: Odd-and branched-chain fatty acids

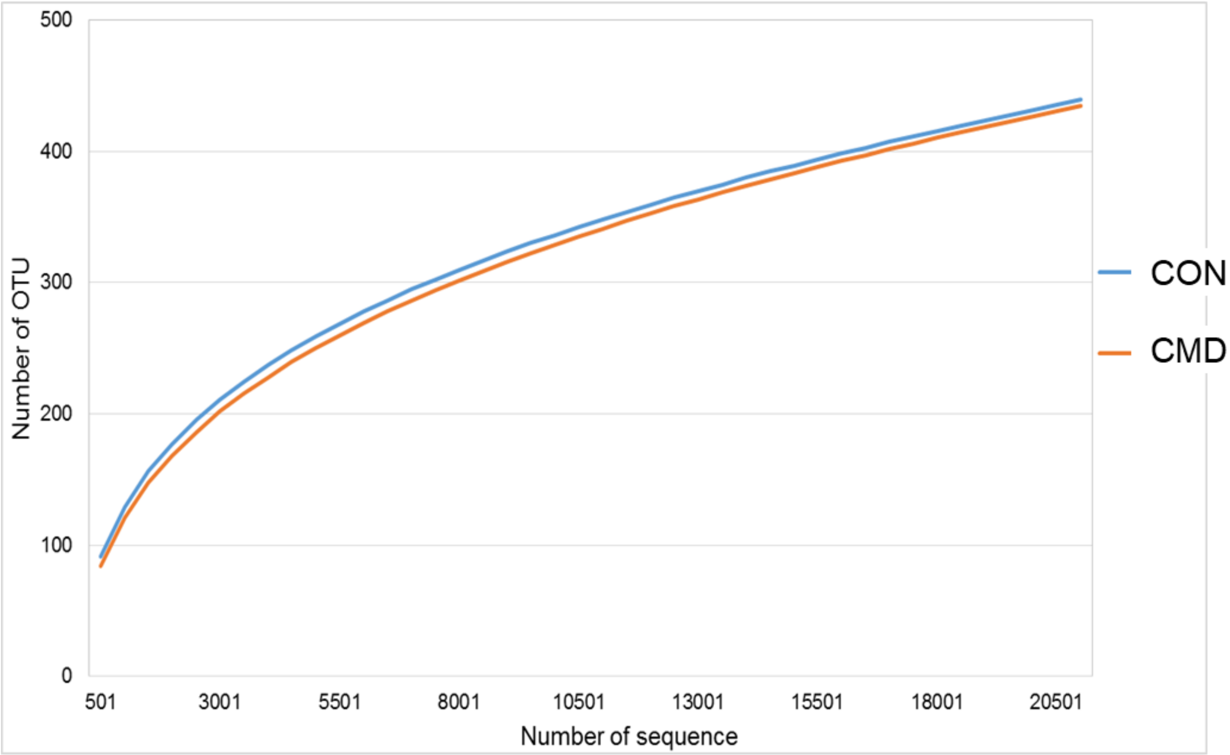

Supplementary Figure S1. Rarefaction curves showing the sequencing depth of the rumen bacterial communities in lambs fed control diet (CON) and cardoon meal diet (CMD).

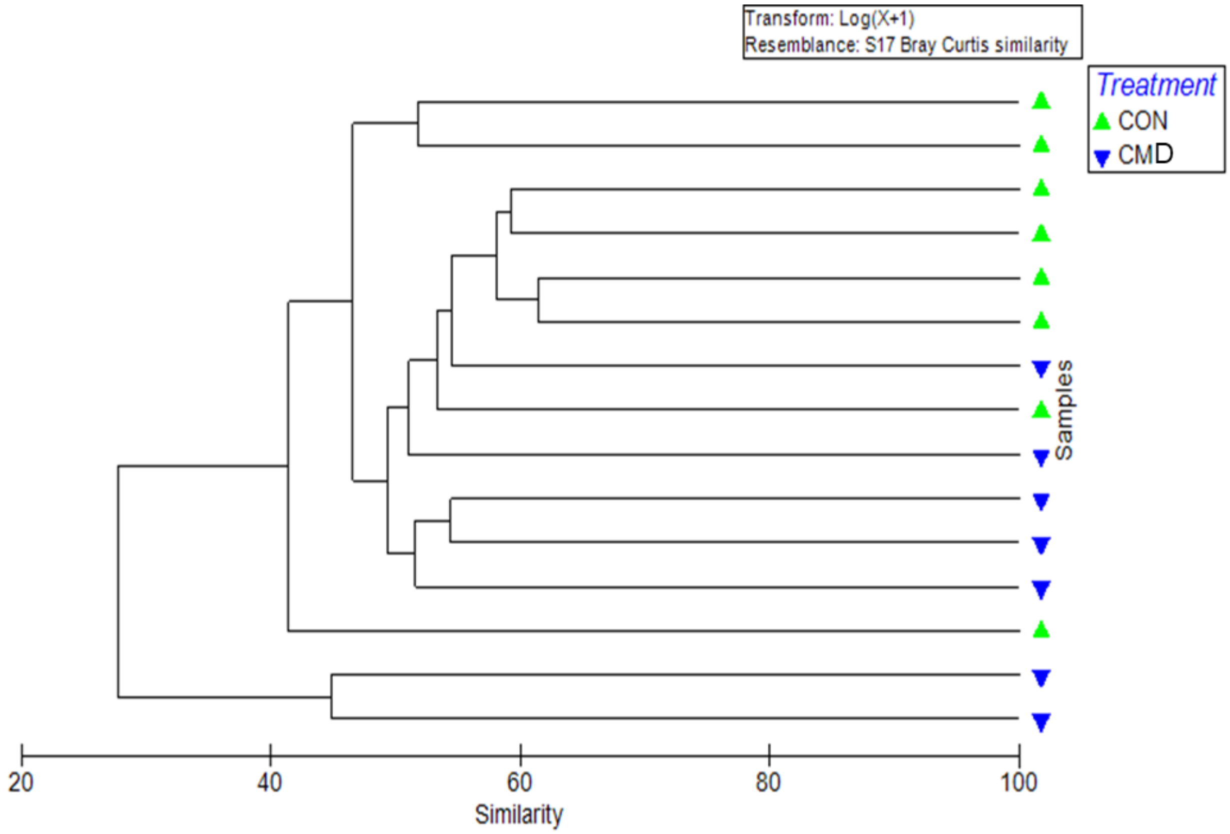

Supplementary Figure S2. Dendrogram plot of hierarchical cluster analysis of rumen samples obtained from lambs fed a control diet (CON) and cardoon meal diet (CMD). Distance between clusters was calculated with group average of resemblance matrix created from log-transformed OTU data sets.

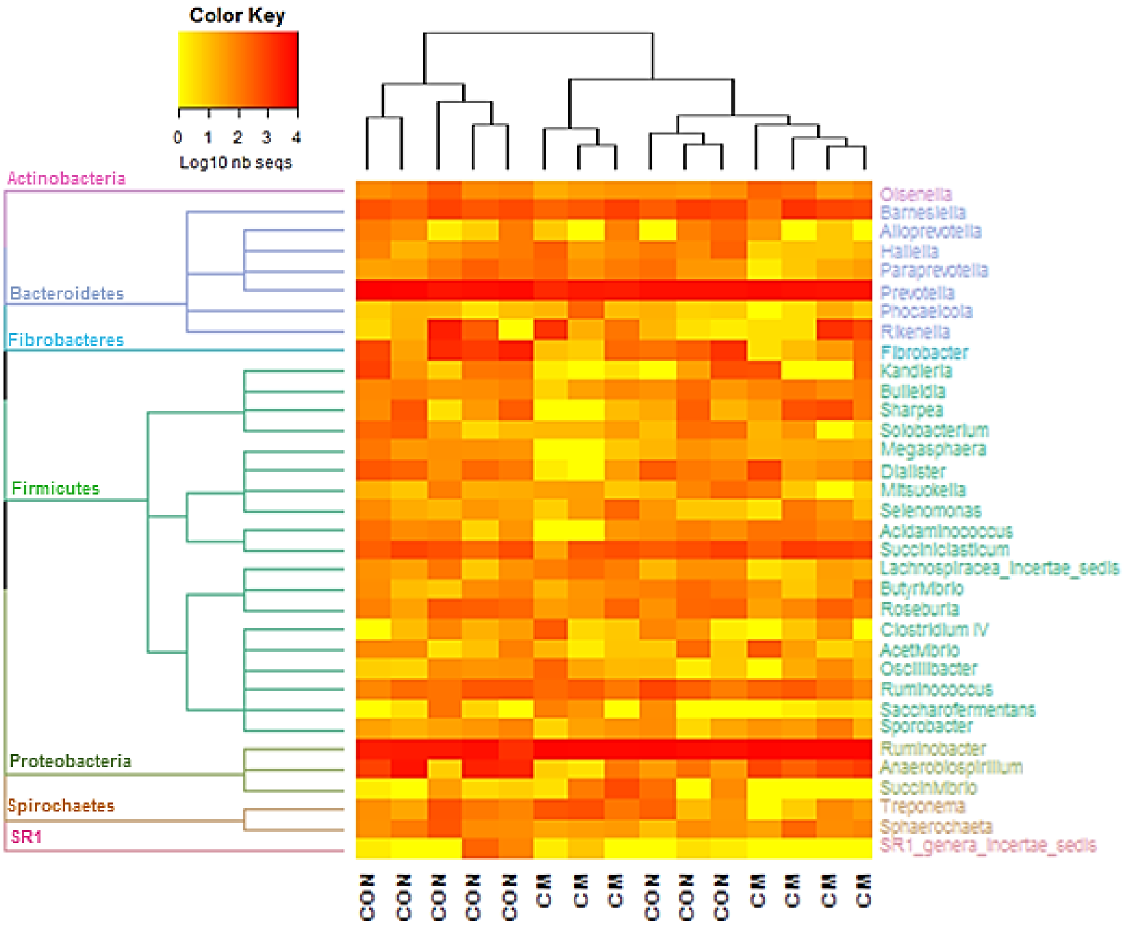

Supplementary Figure S3. Heat map describing the abundance of bacterial genera in the rumen of lambs fed control diet (CON) and cardoon meal diet (CMD). Bacterial genera with relative abundance less than 0.1% were discarded and a dendrogram was created based on UPGMA (Unweighted Pair Group Method with Arithmetic Mean) clustering of the Bray-Curtis distances.

REFERENCES

1 Sylvester, J. T., Karnati, S. K., Yu, Z., Morrison, M. & Firkins, J. L. Development of an assay to quantify rumen ciliate protozoal biomass in cows using real-time PCR. *The Journal of Nutrition* **134**, 3378-3384 (2004).

2 Maeda, H. *et al.* Quantitative real-time PCR using TaqMan and SYBR Green for Actinobacillus actinomycetemcomitans, Porphyromonas gingivalis, Prevotella intermedia, tetQ gene and total bacteria. *FEMS Immunology & Medical Microbiology* **39**, 81-86 (2003).

3 Denman, S. E., Tomkins, N. W. & McSweeney, C. S. Quantitation and diversity analysis of ruminal methanogenic populations in response to the antimethanogenic compound bromochloromethane. *FEMS Microbiology Ecology* **62**, 313-322 (2007).

4 Denman, S. E. & McSweeney, C. S. Development of a real-time PCR assay for monitoring anaerobic fungal and cellulolytic bacterial populations within the rumen. *FEMS microbiology ecology* **58**, 572-582 (2006).

5 Spear, G. T. *et al.* Comparison of the diversity of the vaginal microbiota in HIV-infected and HIV-uninfected women with or without bacterial vaginosis. *Journal of Infectious Diseases* **198**, 1131-1140 (2008).
